# COVID-19 vaccine uptake and intention during pregnancy in Canada

**DOI:** 10.17269/s41997-022-00641-9

**Published:** 2022-04-27

**Authors:** Laura Reifferscheid, Emmanuel Marfo, Ali Assi, Eve Dubé, Noni E. MacDonald, Samantha B. Meyer, Julie A. Bettinger, S. Michelle Driedger, Joan Robinson, Manish Sadarangani, Sarah E. Wilson, Karen Benzies, Samuel Lemaire-Paquette, Arnaud Gagneur, Shannon E. MacDonald

**Affiliations:** 1 Faculty of Nursing, University of Alberta, Edmonton, AB Canada; 2 Institut national de santé publique du Québec, Québec City, QC Canada; 3 Department of Anthropology, Université Laval, Québec City, QC Canada; 4 Department of Pediatrics, Dalhousie University, Halifax, NS Canada; 5 School of Public Health Sciences, University of Waterloo, Waterloo, ON Canada; 6 Vaccine Evaluation Centre, BC Children’s Hospital Research Institute, University of British Columbia, Vancouver, BC Canada; 7 Department of Community Health Sciences, Rady Faculty of Health Sciences, University of Manitoba, Winnipeg, MB Canada; 8 Department of Pediatrics, University of Alberta, Edmonton, AB Canada; 9 Department of Pediatrics, University of British Columbia, Vancouver, BC Canada; 10 ICES, Toronto, ON Canada; 11 Dalla Lana School of Public Health, University of Toronto, Toronto, ON Canada; 12 Public Health Ontario, Toronto, ON Canada; 13 Faculty of Nursing, University of Calgary, Calgary, AB Canada; 14 Centre de Recherche du CHUS, Sherbrooke, QC Canada; 15 Department of Pediatrics, Université de Sherbrooke, Sherbrooke, QC Canada

**Keywords:** COVID-19, Pandemic, Vaccination, Immunization, Pregnancy, Intention, Uptake, COVID-19, pandémie, vaccination, immunisation, grossesse, intention, adoption

## Abstract

**Objective:**

To investigate COVID-19 vaccine uptake and intent among pregnant people in Canada, and determine associated factors.

**Methods:**

We conducted a national cross-sectional survey among pregnant people from May 28 through June 7, 2021 (*n* = 193). Respondents completed a questionnaire to determine COVID-19 vaccine acceptance (defined as either received or intend to receive a COVID-19 vaccine during pregnancy), factors associated with vaccine acceptance, and rationale for accepting/not accepting the vaccine.

**Results:**

Of 193 respondents, 57.5% (*n* = 111) reported COVID-19 vaccine acceptance. Among those who did not accept the vaccine, concern over vaccine safety was the most commonly cited reason (90.1%, *n* = 73), and 81.7% (*n* = 67) disagreed with receiving a vaccine that had not been tested in pregnant people. Confidence in COVID-19 vaccine safety (aOR 16.72, 95% CI: 7.22, 42.39), Indigenous self-identification (aOR 11.59, 95% CI: 1.77, 117.18), and employment in an occupation at high risk for COVID-19 exposure excluding healthcare (aOR 4.76, 95% CI: 1.32, 18.60) were associated with vaccine acceptance. Perceived personal risk of COVID-19 disease was not associated with vaccine acceptance in the multivariate model.

**Conclusion:**

Vaccine safety is a primary concern for this population. Safety information should be communicated to this population as it emerges, along with clear messaging on the benefits of vaccination, as disease risk is either poorly understood or poorly valued in this population.

## Résumé

**Objectif:**

Examiner l’intention de se faire vacciner contre la COVID-19 et l’adoption du vaccin anti-COVID-19 chez les personnes enceintes au Canada, et déterminer les facteurs associés.

**Méthode:**

Du 28 mai au 7 juin 2021, nous avons mené un sondage transversal national auprès de personnes enceintes (*n* = 193). Les répondantes ont rempli un questionnaire pour nous permettre de déterminer l’acceptation du vaccin contre la COVID-19 (définie comme le fait d’avoir reçu ou l’intention de recevoir un vaccin anti-COVID-19 durant la grossesse), les facteurs associés à l’acceptation du vaccin et les motifs d’acceptation ou de refus du vaccin.

**Résultats:**

Sur les 193 répondantes, 57,5 % (*n* = 111) ont dit accepter le vaccin contre la COVID-19. Chez celles qui n’acceptaient pas le vaccin, l’inquiétude face à son innocuité était la raison la plus souvent citée (90,1 %, *n* = 73), et 81,7 % (*n* = 67) n’acceptaient pas de recevoir un vaccin qui n’avait pas été testé sur des personnes enceintes. La confiance en l’innocuité du vaccin anti-COVID-19 (RCa 16,72, IC de 95 % : 7,22, 42,39), l’auto-identification en tant que personne autochtone (RCa 11,59, IC de 95 % : 1,77, 117,18) et l’emploi dans une profession à risque élevé d’exposition à la COVID-19, sauf les soins de santé (RCa 4,76, IC de 95 % : 1,32, 18,60) étaient associés à l’acceptation du vaccin. Le risque personnel perçu de tomber malade de la COVID-19 n’était pas associé à l’acceptation du vaccin dans notre modèle multivarié.

**Conclusion:**

L’innocuité du vaccin est une préoccupation majeure dans cette population. Des informations sur l’innocuité devraient être communiquées aux personnes enceintes au fur et à mesure qu’elles sont connues, ainsi que des messages clairs sur les avantages de la vaccination, car le risque de maladie est soit mal compris, soit déconsidéré dans cette population.

## Introduction

Pregnant people who become infected with the novel severe acute respiratory syndrome coronavirus 2 (SARS-CoV-2) are at increased risk of morbidity and mortality from COVID-19 illness compared to non-pregnant persons (Allotey et al., 2020). Adverse outcomes include preeclampsia and preterm birth (Wei et al., 2021). As of November 2021, over 8500 pregnant people have tested positive for COVID-19 in Canada (Reproductive Infectious Diseases Program, 2021), with reports from some Canadian provinces showing increased hospitalizations and admissions to intensive care units in this population compared to non-pregnant peers (Money et al., 2021).

In Canada, the first COVID-19 vaccine (an mRNA vaccine) became available during the week of December 13, 2020 (Government of Canada, 2021a) but recommendations for vaccine use during pregnancy have varied. Initial guidance from Canada’s National Advisory Committee on Immunization (NACI) on December 12, 2020, advised that pregnant people should not be offered COVID-19 vaccination unless individual benefits were deemed to outweigh risks (Government of Canada, 2021b). One month later, NACI updated its guidelines to recommend offering COVID-19 vaccination to pregnant people, following appropriate counselling to ensure informed decision-making (Government of Canada, 2021b). In April 2021, an observational study of more than 35,000 pregnant people who had received COVID-19 mRNA vaccines indicated no safety concerns (Shimabukuro et al., 2021). On May 3, 2021, NACI published a recommendation indicating pregnant people should preferentially receive a COVID-19 mRNA vaccine (labelled as discretionary, indicating alternative approaches may be reasonable in some circumstances) (Government of Canada, 2021b). This was updated to a strong recommendation on May 28, indicating that clear and compelling evidence is required for an alternative approach (Government of Canada, 2021b).

Given the risk to the population and changing guidelines, it is important to understand COVID-19 vaccine acceptance, both intention to receive the vaccine and vaccine uptake behaviour, among pregnant people in Canada. Research from other high-income countries conducted earlier in the pandemic, before COVID-19 vaccines were widely available, indicated that intention to be vaccinated ranged broadly among pregnant people (Battarbee et al., 2021; Carbone et al., 2021; Ceulemans et al., 2021; Skirrow et al., 2021; Skjefte et al., 2021), likely impacted by both the stage of the pandemic (Ceulemans et al., 2021) and the country of study (Ceulemans et al., 2021; Skjefte et al., 2021). Fewer studies have reported on COVID-19 vaccine uptake among pregnant people, and little is known about COVID-19 vaccine acceptance during pregnancy in Canada. The purpose of this study is to explore COVID-19 vaccine behaviour and intent among pregnant people in Canada.

## Methods

### Survey design and sample

We conducted a national, cross-sectional, web-based survey from May 28 through June 7, 2021, 5 months after COVID-19 vaccines were first approved in Canada, and after NACI had recommended vaccine use during pregnancy. The survey was distributed by an established national commercial polling company (Leger, 2021), with respondents drawn from their panel of > 400,000 Canadians. The sample was representative of Canada’s provincial/territorial population distribution based on the most recent detailed census (Statistics Canada, 2021a). Criteria to participate in the study included (1) ≥ 15 years of age; (2) ability to read either English or French; (3) currently pregnant; and (4) not vaccinated against COVID-19 prior to pregnancy.

To ensure rigour and validity, a unique URL was created for each participant, telephone follow-up with 15% of participants was completed for identity verification, and quality-control questions were included to identify and eliminate inattentive respondents (Eysenbach, 2004). This study was approved by the Health Research Ethics Board of the University of Alberta.

### Survey and measures

Survey questions focussed on vaccine acceptance, factors that influenced acceptance, rationale for vaccine decision, and respondent sociodemographics. Survey items were developed based on published literature, previously validated questions about vaccination intention (Betsch et al., 2018), areas of focus for our policy partners (including the NACI Secretariat), and the expertise of our national team of immunization researchers and policy advisors. Prior to study initiation, the survey was reviewed by three public health experts for content validity and pilot tested for readability and usability by team members as well as a convenience sample of 20 members of the public.

Our outcome variable was COVID-19 vaccine acceptance during pregnancy, defined as vaccine receipt or intention to receive while pregnant. To assess vaccine receipt, we asked respondents whether they had received any doses of COVID-19 vaccine while pregnant. Respondents who indicated they were unvaccinated were then asked to state their intention for receiving the COVID-19 vaccine. Response options included “Yes, while I am pregnant”, “Yes, but I will wait until after my baby is born”, “I am undecided”, and “No”. For analysis, responses were combined into a binary variable: the “Yes” category included those who responded they had received or intended to receive the vaccine while pregnant, with all other answers coded as “No”. In order to test the assumption that respondents who reported receiving a COVID-19 vaccine were similar in their responses to those who reported intention to receive while pregnant (i.e., our combined outcome variable), we conducted a sensitivity analysis, repeating the logistic regression after removing the participants who reported intent to receive while pregnant. Results of the sensitivity analysis indicated no difference between the two groups; thus, only results using the combined outcome variable are reported here.

Exposure variables included sociodemographic characteristics (i.e., age, education level, self-reported ethnicity), self-reported chronic illness, employment occupation and status, and perceptions of COVID-19 disease and vaccine. To determine COVID-19-related perceptions, all respondents were asked to rate their level of agreement with the following statements: “I am completely confident that the COVID-19 vaccines that are available in Canada are safe” (confidence in COVID-19 vaccine safety); “Everyday stress (such as competing priorities or many demands on my time) will prevent me from getting the COVID-19 vaccine” (perceived constraints); and, “I am at risk for becoming sick from COVID-19 disease” (risk of COVID-19 disease). Responses were collected using a 5-point Likert scale and were collapsed into agree (scores of 4 and 5) or disagree/neutral (scores of 1–3) for analysis, based on natural patterns in the data, and common treatment in the literature.

Respondents were asked to identify the main reasons for their vaccine decision. They were asked to rank their top three reasons from a list of choices and were given the option to provide a free-text response to explain their choice. In order to assess the impact of lack of clinical trial data for pregnant people on vaccine acceptance, respondents were asked to rate their level of agreement with receiving a COVID-19 vaccine that had not been tested in pregnant people. Response options were expressed on a 5-point Likert scale, with responses collapsed into agree (scores of 4 and 5), neutral (score of 3), and disagree (scores of 1 and 2). This question was not included in the multivariate analysis; therefore, neutral and disagree responses were reported separately.

### Statistical analysis

Descriptive statistics were calculated for all variables, i.e., frequencies and percentages for categorical variables, and means and standard deviations for continuous variables. We used logistic regression to examine the association between exposure variables and COVID-19 vaccine acceptance. Exposure variables included in the multivariate model were determined a priori based on published literature and vaccine prioritization guidelines. Odds ratios (OR), adjusted odds ratios (aOR), and 95% confidence intervals (CI) were calculated, with statistical significance set at *p* < 0.05. The age variable met the assumption of linearity in the final model, and all variables were confirmed to provide unique information (defined as variance inflation factor < 5). “Prefer not to answer” responses were excluded from the multivariable regression analysis by listwise deletion. Descriptive analysis was completed using SPSS version 26.0 (IBM, Chicago, IL, USA) and logistic regression was completed using R version 4.0.2 (R Foundation, Vienna, AT).

## Results

The national polling company identified 193 individuals in their panel who met our inclusion criteria. Due to the standard recruitment process used by the polling company, the survey response rate was unknown. COVID-19 vaccine acceptance and demographic characteristics of respondents are detailed in Table 1. A total of 111 respondents (57.5%) reported vaccine acceptance (receipt or intent to receive the vaccine during pregnancy). The remaining respondents reported they intended to receive the vaccine after pregnancy (21.8%), were undecided about receiving the vaccine (13.0%), or had no intention of receiving the vaccine (7.8%). Most respondents were from Ontario (42.5%) and Quebec (20.7%). Respondents were provided several gender identity options; all identified as women. The mean age of respondents was 31.0 years (range 18–52 years), similar to the mean maternal age in Canada (31.3 years) (Statistics Canada, 2021c). The majority identified as white (62.7%), and more than half reported some level of university education. More than 60% of respondents indicated an annual household income of > $80,000. A total of 40.4% indicated that they were employed in an occupation that was not high risk for COVID-19 exposure, and a similar proportion of respondents (39.9%) indicated full- or part-time employment in an occupation that was high risk for COVID-19 exposure (healthcare worker or other high-risk occupation). Almost 30% of respondents reported at least one chronic health condition.

**Table 1:** Participant characteristics (*N* = 193)

Variable	*n* (%) or mean (SD)
COVID-19 vaccine receipt and intent	
Received ≥1 dose of vaccine while pregnant^a^	93 (48.2%)
Intends to receive vaccine while pregnant^a^	18 (9.3%)
Intends to receive vaccine after pregnancy	42 (21.8%)
Undecided	25 (13.0%)
No intention of receiving vaccine	15 (7.8%)
Age (years)	31.0 (6.2)
Location of residence	
British Columbia	19 (9.8%)
Alberta	27 (14.0%)
Prairies^b^	15 (7.8%)
Ontario	82 (42.5%)
Quebec	40 (20.7%)
Atlantic provinces^c^	10 (5.2%)
Highest level of education completed	
High school or less	27 (14.0%)
Non-university certificate or diploma (college/apprenticeship)	52 (26.9%)
University certificate, Bachelor’s degree, post-graduate degree	114 (59.1%)
Household income	
< $40,000	17 (8.8%)
$40,000–$79,999	48 (24.9%)
≥$80,000	119 (61.7%)
Prefer not to answer	9 (4.7%)
Self-reported ethnicity	
White	121 (62.7%)
Visible minority or white-visible minority^d^	59 (30.6%)
Indigenous^e^	12 (6.2%)
Prefer not to answer	1 (0.5%)
Employment status	
Employed, healthcare worker^f^	29 (15.0%)
Employed, other high-risk occupation^g^	48 (24.9%)
Employed, not high risk^h^	78 (40.4%)
Unemployed	34 (17.6%)
Prefer not to answer	4 (2.1%)
One or more chronic health conditions^i^	
Yes	53 (27.5%)
No	140 (72.5%)

*SD*, standard deviation

^a^Combined to produce outcome variable (vaccine acceptance)

^b^Saskatchewan and Manitoba

^c^Nova Scotia, New Brunswick, Prince Edward Island, and Newfoundland and Labrador

^d^Self-identified as visible minority groups (e.g., Black, Latin/Central American, Arabic/West Asian/North African, East Asian, South Asian, Other) as per Statistics Canada (2021). Visible minorities are defined as non-white and non-Indigenous (Statistics Canada, 2021b)

^e^Self-identified as First Nations, Métis, or Inuk

^f^Full-time (35+ h/week) or part-time (< 35 h/week) employment as a healthcare worker, personal care worker, or caregiver providing care in a long-term care facility or other care facility for seniors

^g^Full- or part-time employment as either of: non-healthcare worker essential in managing the COVID-19 response to providing frontline care for COVID-19 patients; worker contributing to the maintenance of other essential services for the functioning of society (i.e., those who cannot work virtually and have increased exposure to COVID-19 (e.g., police, firefighters, grocery store staff, school staff))

^h^Full- or part-time employment in occupation that does not fit into any of the above categories

^i^Pre-existing chronic conditions defined as severe asthma requiring medical follow-up or hospitalization, other severe chronic lung disease requiring regular medical follow-up or hospitalization (e.g., emphysema, chronic bronchitis, or cystic fibrosis), severe heart problem requiring regular medical follow-up or hospitalization (e.g., angina, heart failure, heart attack), diabetes, liver disease, chronic kidney disease, cancer or other immune system disorder, immunocompromised state from organ transplant or immune deficiencies, obesity, dementia

**Fig. 1:**
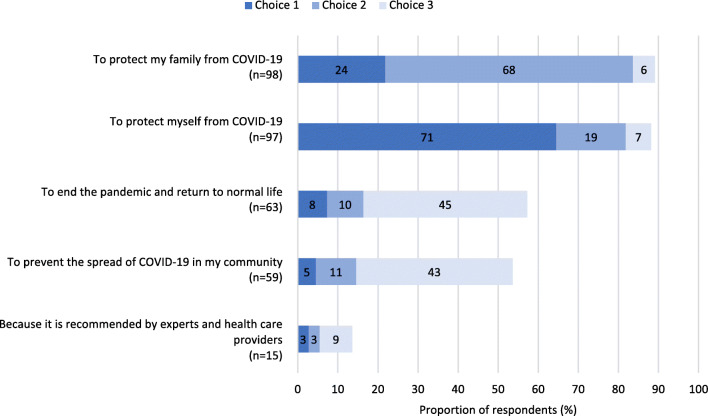
Main reasons for COVID-19 vaccine acceptance for the respondents who received or intended to receive the vaccine during pregnancy (*N* = 110). A total of four free-text answers were received. Three were reclassified into the presented choices and one was discarded as not relevant to the question

**Fig. 2:**
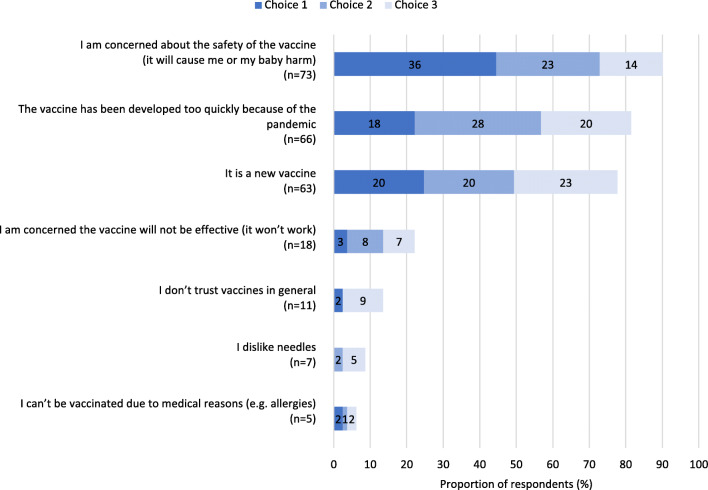
Main reasons for being reluctant or undecided about receiving the COVID-19 vaccine for respondents who did not receive or intend to receive the vaccine during pregnancy (*N* = 81). A total of five free-text answers were received. Four were reclassified into the presented choices and one was discarded as not relevant to the question

Figure 1 shows the top 3 reasons for accepting the vaccine for respondents who received or intended to receive the vaccine during pregnancy. The majority of respondents cited their desire to protect their family (89.1%) or themselves from COVID-19 disease (88.2%) as motivating factors. Only 13.6% (*n* = 15) of respondents identified recommendation by experts and healthcare providers as a top 3 reason for receiving a vaccine. The most common reason for not accepting COVID-19 vaccination during pregnancy was a concern about the safety of the vaccine, for either self or fetus (90.1%, *n* = 73; Fig. 2). Eleven respondents (13.6%) indicated they did not trust vaccines in general.

**Fig. 3:**
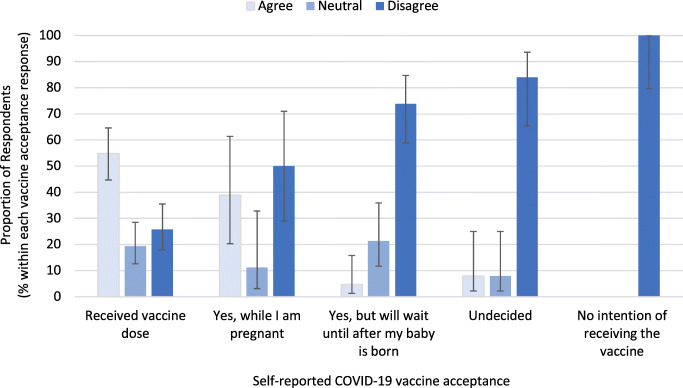
Respondents’ agreement with receiving a vaccine that had not been tested in pregnant people, by self-reported COVID-19 vaccine acceptance (*N* = 193)

Of the 193 respondents, 32.1% (*n* = 62) reported that they would be willing to receive a COVID-19 vaccine that had not been tested in pregnant people. Most of these respondents (93.5%, *n* = 58) reported vaccine acceptance, representing 54.8% (*n* = 51) of those who reported they had already received a COVID-19 vaccine while pregnant, and 38.9% (*n* = 7) of those planning to receive a COVID-19 vaccine while pregnant (Fig. 3). Approximately 51.8% of respondents (*n* = 100) indicated they disagreed with receiving a vaccine that had not been tested in pregnant people; 81.7% (*n* = 67) of these people reported they did not accept COVID-19 vaccination while pregnant. Those who disagreed with receiving a vaccine that had not been tested in pregnant people made up 73.8% (*n* = 31) of the group who plan to receive the COVID-19 vaccine after pregnancy, 84.0% (*n* = 21) of those undecided about receiving the COVD-19 vaccine, and 100% (*n* = 15) of those with no intention to receive a COVID-19 vaccine.

Several factors were associated with COVID-19 vaccine acceptance (Table 2). In the initial unadjusted results, employment in any occupation, concern over risk of illness from COVID-19 disease, and confidence in the safety of available COVID-19 vaccines were associated with vaccine acceptance. In the multivariate analysis, only Indigenous self-identification (compared to white self-identification; aOR 11.59, 95% CI: 1.77, 117.18), employment in an occupation at high risk for COVID-19 exposure other than healthcare (compared to unemployed; aOR 4.76, 95% CI: 1.32, 18.60), and confidence in COVID-19 vaccine safety (compared to neutral/disagree; aOR 16.72, 95% CI: 7.22, 42.39) remained significant. Sociodemographic characteristics (age, education, household income), self-reported chronic illness, and perceived access constraints were not significantly associated with vaccine acceptance in either the univariate or the multivariate regression.

**Table 2:** Unadjusted and adjusted logistic regression of factors associated with COVID-19 vaccine acceptance during pregnancy

Variable	COVID-19 vaccine acceptance, *n* (%)^a^	COVID-19 vaccine non-acceptance, *n* (%)^a^	Unadjusted OR (95% CI)	Adjusted^b^ OR (95% CI)
Age in years	N/A	N/A	1.01 (0.96, 1.06)	0.94 (0.88, 1.01)
Highest level of education completed				
High school or less	14 (51.9)	13 (48.1)	Ref	Ref
Non-university certificate or diploma	21 (40.4)	31 (59.6)	0.63 (0.24, 1.60)	0.27 (0.06, 1.06)
University certificate, Bachelor’s degree, post-graduate degree	76 (66.7)	38 (33.3)	1.86 (0.79, 4.37)	1.02 (0.28, 3.57)
Household income				
< $40,000	9 (52.9)	8 (47.0)	Ref	Ref
$40,000–$79,999	20 (41.7)	28 (58.3)	0.63 (0.20. 1.94)	0.68 (0.14, 3.16)
≥ $80,000	75 (63.0)	44 (37.0)	1.52 (0.53, 4.24)	1.39 (0.31, 6.24)
Self-identified ethnicity				
White	69 (57.0)	52 (43.0)	Ref	Ref
Visible minority or white-visible minority	31 (52.5)	28 (47.5)	0.83 (0.45, 1.56)	0.68 (0.27, 1.66)
Indigenous	10 (83.3)	2 (16.7)	3.77 (0.94, 25.20)	11.59 (1.77, 117.18)
Employment status				
Unemployed	12 (35.3)	22 (64.7)	Ref	Ref
Employed, not high risk	45 (57.7)	33 (42.3)	2.50 (1.10, 5.90)	1.88 (0.58, 6.42)
Employed, other high-risk occupation	33 (68.8)	15 (31.2)	4.03 (1.62, 10.53)	4.76 (1.32, 18.60)
Employed, healthcare worker	19 (65.5)	10 (34.5)	3.48 (1.26, 10.19)	2.83 (0.69, 12.34)
One or more chronic health conditions				
No	84 (60.0)	56 (40.0)	Ref	Ref
Yes	27 (50.9)	26 (49.1)	0.69 (0.37, 1.31)	0.78 (0.31, 1.93)
Risk of COVID-19 disease				
Neutral/disagree	43 (48.3)	46 (51.7)	Ref	Ref
Agree	68 (65.4)	36 (34.6)	2.02 (1.14, 3.63)	1.11 (0.47, 2.56)
Confidence in COVID-19 vaccine safety				
Neutral/disagree	24 (27.3)	64 (72.7)	Ref	Ref
Agree	87 (82.9)	18 (17.1)	12.89 (6.59, 26.38)	16.72 (7.22, 42.39)
Perceived constraints				
Neutral/disagree	29 (47.5)	32 (52.4)	Ref	Ref
Agree	82 (62.1)	50 (37.9)	1.81 (0.98, 3.36)	0.80 (0.30, 2.08)

^a^Percent of population subgroup

^b^Adjusted for all other variables in the table

## Discussion

We conducted a national, cross-sectional survey to explore COVID-19 vaccine uptake behaviour and intention among pregnant people. A total of 193 individuals participated in our survey from May 28 through June 7, 2021, after COVID-19 vaccination during pregnancy had been recommended by NACI.

We found that most pregnant people (57.5%) had received (48.2%) or intended to receive (9.3%) the COVID-19 vaccine while pregnant. These results are within the wide range of COVID-19 vaccine intention for pregnant people reported in the literature (28–62%), including results from the United Kingdom (62.1%; August–October 2020) (Skirrow et al., 2021), the United States (41%; August–December 2020) (Battarbee et al., 2021), Italy (28.2%; January 2021) (Carbone et al., 2021), and multinationally (61.4%; April–July 2020) (Ceulemans et al., 2021), (52.0%; October–November 2020) (Skjefte et al., 2021). Our results are somewhat higher than COVID-19 vaccine uptake results reported elsewhere. In a US study, only 16.3% of all pregnant people had received the COVID-19 vaccine between December 14 and May 8, 2021, though monthly coverage steadily increased during the study period (Razzaghi et al., 2021). Results from Ontario show a similar trend, with vaccine uptake increasing from approximately 0.02% in December 2020 to 38.1% in May 2021 (Better Outcomes Registry & Network Ontario, 2021). These studies used administrative data to determine vaccine uptake; in contrast, our study relied on self-report, which may overestimate coverage in this population (Poliquin et al., 2019).

Confidence in vaccine safety was the most significant predictor of COVID-19 vaccine acceptance among respondents in our study, and vaccine safety concerns were the most cited reason for not accepting the COVID-19 vaccine during pregnancy. Similar results have been reported in the literature (Battarbee et al., 2021; Carbone et al., 2021; Skjefte et al., 2021), with studies conducted earlier in the pandemic identifying a lack of pregnancy-specific safety information as a barrier to positive vaccine intention (Skirrow et al., 2021). Vaccine safety concerns are almost universally reported as a barrier to vaccine uptake during pregnancy, for both routine and pandemic vaccinations (Poliquin et al., 2019). Our results indicate that a majority of those who did not accept the vaccine during pregnancy disagreed with receiving a vaccine that had not been tested in pregnant people. However, over half of those who did not accept vaccination during pregnancy reported that they planned to receive the COVID-19 vaccine after giving birth, and few indicated a negative perception of vaccines in general. These findings support the necessity of pregnancy-specific safety information about the COVID-19 vaccine for optimizing vaccine uptake in this population. Notably, a lack of pregnancy-specific information also appears to be a concern among those who reported they had already received a COVID-19 vaccine dose during pregnancy. It will be important to explore whether those who received the vaccine were simply unaware of the lack of COVID-19 vaccine safety information early in vaccine rollout, or whether emerging safety information later in the pandemic informed their vaccination decision. Regardless, in an environment where safety information is evolving, it is important that pregnant people are provided with ongoing opportunities to discuss vaccine safety, and that care providers have adequate information and techniques to support and communicate individual risk-benefit assessment.

Self-protection was identified by almost 65% of respondents as their primary reason (choice 1) for accepting a COVID-19 vaccine. Motivations outside of self (protect family, protect community) were also common. Despite this, perceived personal risk was not a predictor of vaccine acceptance in the multivariate analysis, suggesting that respondents who did not accept the vaccine during pregnancy may have had a higher risk perception of the vaccine than the disease. In addition, despite the established association between chronic illness and negative COVID-19 outcomes (Public Health Agency of Canada, 2021), presence of a chronic medical condition was not associated with increased vaccine acceptance in our study. Other literature reports mixed results on the importance of personal risk in COVID-19 vaccine intentions (Battarbee et al., 2021), with one study identifying perceived personal risk as less important than general concern about COVID-19 (Skjefte et al., 2021). We did not assess whether respondents were aware of the elevated risk for negative COVID-19 outcomes during pregnancy, though given that knowledge in this area is still developing, it is likely that lack of disease risk awareness plays a role in COVID-19 vaccine acceptance. In the general adult population, risk-based messaging about COVID-19 disease appears to have little impact on those who have concerns about vaccine safety and efficacy (Motta et al., 2021). Similar results have been found regarding established vaccines in pregnant people, though messaging that emphasizes protective benefit of vaccination to the fetus may be particularly effective (Ellingson et al., 2019).

In our study, employment status was significantly related to COVID-19 vaccine acceptance. Both respondents who work in the healthcare field and those who work in other occupations at higher risk for COVID-19 exposure were more likely to report COVID-19 vaccine acceptance when compared to those who were unemployed. However, employment as a healthcare worker was not statistically significant in the multivariate model. Published literature indicates mixed results on the relationship between COVID-19 vaccine intention and healthcare employment (Battarbee et al., 2021; Ceulemans et al., 2021; Skjefte et al., 2021). It is possible that early prioritization of healthcare workers may have actually hindered COVID-19 vaccine uptake among pregnant healthcare workers, as these individuals may have made decisions about vaccination very early, when little information on COVID-19 vaccine safety during pregnancy was available. However, the specific healthcare-related professions of respondents may have also impacted our results, as significant variation between different healthcare professions has been found for both COVID-19 vaccine intention (Ciardi et al., 2021) and uptake (Dzieciolowska et al., 2021).

Respondents who self-identified as Indigenous were significantly more likely to report COVID-19 vaccine acceptance compared to those who identified as white in this study. Our results also indicate that COVID-19 vaccine acceptance among visible minorities was not significantly different from vaccine acceptance among white respondents. Minority race/ethnicity has been identified as a factor in lower COVID-19 vaccine intention among pregnant people in the UK (Skirrow et al., 2021), and both intention (Battarbee et al., 2021) and uptake (Razzaghi et al., 2021) in the USA; however, the impact of efforts to prioritize vaccine access among these communities is unknown. In Canada, national recommendations identified both Indigenous adults and adults in racialized and marginalized communities as priority groups for early access to COVID-19 vaccines, based on potential increased risk of exposure and/or negative health outcomes (Government of Canada, 2021c). Early in the pandemic, public health campaigns and education strategies developed by Indigenous communities, and grounded in Indigenous tradition, appeared to contribute to initially low COVID-19 case and mortality rates (Richardson & Crawford, 2020). The impact of these efforts on vaccine uptake, in combination with early prioritization, should be explored.

Sociodemographic factors such as age, income, and education were not significantly related to vaccine acceptance in our study. Similar results have been found regarding intention to receive COVID-19 vaccine while pregnant (Battarbee et al., 2021; Carbone et al., 2021), though results may be country-dependent (Skirrow et al., 2021; Skjefte et al., 2021). In contrast, older maternal age, higher income, and increased education have been consistently associated with higher seasonal and pandemic influenza vaccine uptake during pregnancy (Poliquin et al., 2019; Yuen & Tarrant, 2014). Reasons for these relationships have not been well explored, though vaccine access constraints are assumed to play a role (Liu et al., 2012). Efforts to facilitate vaccination during the COVID-19 vaccine rollout may have contributed to more equitable vaccination access, thus minimizing the impact of sociodemographic factors on vaccine uptake. Given this study’s small sample size, these relationships should be explored among larger samples, with a focus on understanding the impact of different vaccination strategies on equitable access.

Vaccine recommendation by a healthcare provider is extensively discussed as an important facilitator for vaccine uptake during pregnancy (Poliquin et al., 2019; Yuen & Tarrant, 2014). In our study, healthcare provider recommendation was cited as a top 3 reason for vaccine acceptance by less than 14% of respondents. Although most respondents did not identify recommendations from healthcare providers as reasons for vaccine acceptance, this may indicate that in the context of pandemic disruption, and significant public education and vaccination efforts, pregnant people who were generally positive about vaccination had less need for healthcare provider input. Evolving national recommendations and an initial lack of pregnancy-specific information on vaccine safety and effectiveness likely also impacted the willingness of maternal care providers to encourage COVID-19 vaccination for their patients (Deruelle et al., 2021). In light of the significant role vaccine safety concerns play, and indications that risk is either poorly understood or undervalued, future research should explore what patient factors determine whether healthcare provider recommendation outweighs safety concerns.

### Strengths and limitations

Our study captured both COVID-19 vaccine uptake and intention among a national sample of pregnant people at a time when national guidelines recommended that COVID-19 vaccine be offered to all pregnant people. However, the small sample size and use of a pre-existing panel of individuals may limit the generalizability of our results to the broader population of pregnant women in Canada. Reliance on self-reported vaccine uptake is a potential limitation, subject to both recall and social desirability bias. However, the high-profile nature of COVID-19 vaccination means respondents were unlikely to be impacted by recall, and the online and anonymous nature of the survey likely decreases the impact of social desirability on responses. The survey excluded pregnant individuals who were vaccinated prior to pregnancy, who may have been more likely to accept the vaccine during their pregnancy. This may have resulted in an underestimation of COVID-19 vaccine acceptance, though is more likely to be a factor among groups who were prioritized very early in the vaccine rollout (e.g., healthcare workers, Indigenous peoples). Although national vaccine recommendations provide some indication of how vaccines were prioritized in Canada, program implementation decisions, including sequencing of prioritized groups, occurs at the provincial/territorial level and thus was likely subject to regional variation. Given the small sample size, we were unable to assess any regional variation in COVID-19 vaccine acceptance. Finally, the cross-sectional design of the study precludes our ability to identify any trends in vaccine acceptance with time. Given the rapidly evolving conditions of the COVID-19 pandemic, and associated vaccine information and policy, COVID-19 vaccine acceptance among this population will be subject to change.

## Conclusion

In light of the evolving nature of COVID-19 vaccine safety information for this population group, it will be important to share information as it becomes available. Additional information about disease risks and vaccine safety specific to pregnancy may result in increased uptake among pregnant people, though only if used in concert with continued efforts to facilitate vaccination access. More research is required to understand the impact of prioritization for vaccination on disease risk perception, vaccine safety knowledge, and vaccine uptake. In addition, it will be important to clarify healthcare providers’ role in vaccination decision-making, particularly among those who are unsure about vaccine receipt.

## Contributions to knowledge

What does this study add to existing knowledge?

• Previous studies have reported on COVID-19 vaccine intention among pregnant people; our study is among the first to explore vaccination behaviour in this population.

• Lower socioeconomic status and non-white self-reported ethnicity were not associated with lower vaccine acceptance in this study; public health and community efforts to encourage uptake and facilitate access may have contributed to higher uptake in these populations.

• Lack of pregnancy-specific COVID-19 vaccine safety data has been highlighted as a significant concern.

What are the key implications for public health interventions, practice, or policy?

• Safety of the COVID-19 vaccine is a primary concern for pregnant people when deciding to receive the vaccine. As pregnant people were not included in initial clinical trials, it is important that safety information is communicated to this population as it emerges.

• Safety information should be accompanied by information about the benefits of vaccination during pregnancy, as the elevated risk of COVID-19 disease in pregnancy is either poorly understood or undervalued.

## Data Availability

Data specific to this manuscript can be requested from the senior author, provided any such requests are covered under the existing ethics approval.

## Code availability

The analysis code can be requested from the senior author, provided any such requests are covered under the existing ethics approval.

## References

[CR1] Allotey J, Stallings E, Bonet M, Yap M, Chatterjee S, Kew T (2020). Clinical manifestations, risk factors, and maternal and perinatal outcomes of coronavirus disease 2019 in pregnancy: Living systematic review and meta-analysis. BMJ.

[CR2] Battarbee, A. N., Stockwell, M. S., Varner, M., Newes-Adeyi, G., Daugherty, M., Gyamfi-Bannerman, C., et al. (2021). Attitudes toward COVID-19 illness and COVID-19 vaccination among pregnant women: A cross-sectional multicenter study during August-December 2020. *Am J Perinatol*. 10.1055/s-0041-1735878 10.1055/s-0041-1735878 34598291

[CR3] Betsch, C., Schmid, P., Heinemeier, D., Korn, L., Holtmann, C., & Böhm, R. ( 2018). Beyond confidence: Development of a measure assessing the 5C psychological antecedents of vaccination. *PLOS ONE, 13*, e0208601. 10.1371/journal.pone.0208601 10.1371/journal.pone.0208601 PMC6285469 30532274

[CR4] Better Outcomes Registry & Network (BORN) Ontario. (2021). COVID-19 vaccination during pregnancy in Ontario: Surveillance report #1, Reporting Interval December 14, 2020 to May 31, 2021. Available at: https://www.bornontario.ca/en/whats-happening/resources/Documents/COVID-19-Vaccination-During-Pregnancy-in-Ontario-Report-1%2D%2D-FINAL.pdf

[CR5] Carbone L, Mappa I, Sirico A, Di Girolamo R, Saccone G, Di Mascio D (2021). Pregnant women’s perspectives on severe acute respiratory syndrome coronavirus 2 vaccine. American Journal of Obstetrics & Gynecology MFM.

[CR6] Ceulemans M, Foulon V, Panchaud A, Winterfeld U, Pomar L, Lambelet V (2021). Vaccine willingness and impact of the COVID-19 pandemic on women’s perinatal experiences and practices—A multinational, cross-sectional study covering the first wave of the pandemic. International Journal of Environmental Research and Public Health.

[CR7] Ciardi F, Menon V, Jensen JL, Shariff MA, Pillai A, Venugopal U (2021). Knowledge, attitudes and perceptions of COVID-19 vaccination among healthcare workers of an inner-city hospital in New York. Vaccines.

[CR8] Deruelle, P., Couffingnal, C., Sibiude, J., Vivanti, A. J., Anselem, O., Luton, D., et al. (2021). Prenatal care providers’ perceptions of the SARS-CoV-2 vaccine for themselves and for pregnant women. *PLOS ONE, 16*(9), e0256080. 10.1371/journal.pone.0256080 10.1371/journal.pone.0256080 PMC8437278 34516551

[CR9] Dzieciolowska S, Hamel D, Gadio S, Dionne M, Gagnon D, Robitaille L (2021). COVID-19 vaccine acceptance, hesitancy, and refusal among Canadian healthcare workers: A multicenter survey. American Journal of Infection Control.

[CR10] Ellingson MK, Dudley MZ, Limaye RJ, Salmon DA, O’Leary ST, Omer SB (2019). Enhancing uptake of influenza maternal vaccine. Expert Review of Vaccines.

[CR11] Eysenbach G (2004). Improving the quality of web surveys: The checklist for reporting results of internet E-Surveys (CHERRIES). J Med Internet Res.

[CR12] Government of Canada. (2021a). Canada COVID-19 vaccination coverage report*.* https://health-infobase.canada.ca/covid-19/vaccination-coverage/. Accessed 15 June 2021.

[CR13] Government of Canada. (2021b). Table of updates: Recommendations on the use of COVID-19 vaccines. https://www.canada.ca/en/public-health/services/immunization/national-advisory-committee-on-immunizationnaci/recommendations-use-covid-19-vaccines/table-updates.html. Accessed 4 August 2021.

[CR14] Government of Canada. (2021c). Guidance on the prioritization of key populations for COVID-19 immunization. .https://www.canada.ca/en/public-health/services/immunization/national-advisory-committee-on-immunization-naci/guidanceprioritization-key-populations-covid-19-vaccination.html Accessed 4 August 2021.

[CR15] Leger Marketing Inc. *We know Canadians*. https://leger360.com/. Accessed 30 May 2021.

[CR16] Liu N, Sprague AE, Yasseen AS, Fell DB, Wen S-W, Smith GN, Walker MC (2012). Vaccination patterns in pregnant women during the 2009 H1N1 influenza pandemic: A population-based study in Ontario, Canada. Canadian Journal of Public Health.

[CR17] Money, D., Elwood, C., Ting, J., Roberts, A., Albert, A., McMclymont, E., et al. (2021). Canadian surveillance of COVID-19 in pregnancy: Epidemiology, maternal, and infant outcomes. Report #4. Available at: https://med-fom-ridprogram.sites.olt.ubc.ca/files/2021/10/CANCOVID_Preg-report-4-19oct2021.pdf

[CR18] Motta M, Sylvester S, Callaghan T, Lunz-Trujillo K (2021). Encouraging COVID-19 vaccine uptake through effective health communication. Frontiers in Political Science.

[CR19] Poliquin V, Greyson D, Castillo E (2019). A systematic review of barriers to vaccination during pregnancy in the Canadian context. Journal of Obstetrics and Gynaecology Canada.

[CR20] Public Health Agency of Canada. (2021). An Advisory Committee Statement (ACS) National Advisory Committee on Immunization (NACI): Recommendations on the use of COVID-19 vaccines. Available at: https://www.canada.ca/content/dam/phac-aspc/documents/services/immunization/national-advisory-committee-on-immunization-naci/recommendations-use-covid-19-vaccines/recommendations-use-covid-19-vaccines-en.pdf

[CR21] Razzaghi H, Meghani M, Pingali C, Crane B, Naleway A, Weintraub E (2021). COVID-19 vaccination coverage among pregnant women during pregnancy: Eight integrated health care organizations, United States, December 14, 2020–May 8, 2021. Morbidity and Mortality Weekly Report.

[CR22] Reproductive Infectious Diseases Program, University of British Columbia. (2021). Canadian Surveillance of COVID-19 in Pregnancy: Epidemiology, maternal and infant outcomes – (as of November 2021). Available at: https://ridprogram.med.ubc.ca/cancovid-preg/

[CR23] Richardson L, Crawford A (2020). COVID-19 and the decolonization of Indigenous public health. CMAJ.

[CR24] Shimabukuro TT, Kim SY, Myers TR, Moro PL, Oduyebo T, Panagiotakopoulos L (2021). Preliminary findings of mRNA COVID-19 vaccine safety in pregnant persons. N Engl J Med.

[CR25] Skirrow, H., Barnett, S., Bell, S., Riaposova, L., Mounier-Jack, S., Kampmann, B., & Holder, B. (2022). Women’s views on accepting COVID-19 vaccination during and after pregnancy, and for their babies: A multi-methods study in the UK. *BMC Pregnancy Childbirth 22,* 33. 10.1101/2021.04.30.21256240. Accessed 04 August 2021. 10.1186/s12884-021-04321-3 PMC8759061 35030996

[CR26] Skjefte M, Ngirbabul M, Akeju O, Escudero D, Hernandez-Diaz S, Wyszynski DF, Wu JW (2021). COVID-19 vaccine acceptance among pregnant women and mothers of young children: Results of a survey in 16 countries. European Journal of Epidemiology.

[CR27] Statistics Canada. (2021a, Feb 08). *Data products, 2016 Census.* https://www12.statcan.gc.ca/census-recensement/2016/dp-pd/index-eng.cfm.

[CR28] Statistics Canada. (2021b, March 25). *Visible minority of person*. https://www23.statcan.gc.ca/imdb/p3Var.pl?Function=DEC&Id=45152. Accessed 4 August 2021.

[CR29] Statistics Canada. (2021c, September 29). *Mean age of mother at time of delivery (live births).* https://www150.statcan.gc.ca/t1/tbl1/en/tv.action?pid=1310041701.

[CR30] Wei SQ, Bilodeau-Bertrand M, Liu S, Auger N (2021). The impact of COVID-19 on pregnancy outcomes: A systematic review and meta-analysis. CMAJ.

[CR31] Yuen CYS, Tarrant M (2014). Determinants of uptake of influenza vaccination among pregnant women - A systematic review. Vaccine.

